# Supplementary material for: Structure‐Based Demystification of Radical Catalysis by a Coenzyme B12 Dependent Enzyme—Crystallographic Study of Glutamate Mutase with Cofactor Homologues
Source: Angew Chem Weinheim Bergstr Ger. 2022 Jul 21;134(35):e202208295. doi: 10.1002/ange.202208295 (PMC10947579; doi:10.1002/ange.202208295)
Supplement: Supplementary file 1 — Supporting Information [file ANGE-134-0-s001.pdf]

Angewandte Chemie
Eine Zeitschrift der Gesellschaft Deutscher Chemiker

Supporting Information

Structure-Based Demystification of Radical Catalysis by a Coenzyme B_{12} Dependent Enzyme—Crystallographic Study of Glutamate Mutase with Cofactor Homologues

K. Gruber, V. Csitkovits, A. Łyskowski, C. Kratky, B. Kräutler**

Supplementary Information

Table of Contents

Experimental Part 2
Materials and Methods 2
X-ray crystallography 2
Supplementary Tables 3
Supplementary Figures 5
Supplementary References 19

Experimental Part

Materials and Methods

5’-Deoxy-5’-adenosylmethyl-cob(III)alamin (AdoMeCbl) and 7’[5’-deoxy-5’-ethyladenosyl]-cob(III)alamin (AdoEtCbl), also named homocoenzyme B_{12} and bis-homocoenzyme B_{12}, respectively, were prepared and crystallised as published.[1] Glutamate mutase (GM) was from *Clostridium cochlearium*.[2]

X-ray crystallography

Glutamate mutase (GM) from *Clostridium cochlearium* was reconstituted with AdoMeCbl and AdoEtCbl, the two ‘stretched’ homologues of coenzyme B_{12},[1] purified and crystallised as described previously.[2] We obtained crystals with sizes of about 0.2 x 0.2 x 0.05 mm using the sitting drop method by mixing 4 μl drops containing 3.5 mg/ml protein, 0.5 mg/ml AdoMeCbl or AdoEtCbl, and 2 mM $CdCl_2$ with 4 μl reservoir solution containing 6% (w/v) PEG-4000 in 0.1 M DL-tartrate at pH=4.5. Purification and crystallisation were performed under red light to prevent photolytic degradation. This precaution was somewhat relaxed for the frozen crystals.[2c] Diffraction data were collected from flash-cooled crystals (cryoprotected with 30% glycerol) at the beamline BW7B of the EMBL outstation at DESY/Hamburg. The data were processed using the XIA2 package[3] employing the programs MOSFLM,[4] XDS,[5] AIMLESS/POINTLESS,[6] and other programs from the CCP4 package.[7]

The crystal structures were solved using the isomorphous structure of GM reconstituted with cyanocob(III)alamin (PDB-code 1CCW)[2b] devoid of all cofactor, ligand, and solvent atoms. After rigid-body refinement, unequivocal residual electron density was observed in both cases for the Cbl-moiety, including the upper 5’-adenosylmethyl and 5’-adenosylethyl ligands (see **Figure S4**) and a stereo-specifically incorporated (*S*,*S*)-tartrate ion occupying the substrate binding site close to the Cbl. The structures were rebuilt and refined using PHENIX[8] and COOT.[9] R_{free}-values[10] were computed from 5% randomly chosen reflections not used for the refinement. Water molecules were automatically detected in difference electron density maps and retained or rejected based on geometric criteria, refined B-factors ($B<50$ Å^2), and map correlation coefficients.

The electron density in the cofactor regions indicated an intact cobalt carbon bond in all B_{12}-molecules. To prevent unwanted van-der-Waals repulsion between these two atoms during structure refinement, an additional geometric restraint was specified for the Co-C bond with an ideal value of 2.0 Å but a large standard deviation of 1.0 Å, thereby allowing an unbiased estimate of its length. We employed the same strategy for treating the interaction between the cobalt atom and the imidazole ring of H16 from the small σ-subunit. Throughout the refinement, the structures were validated using the program Molprobity.[11]

Data on the diffraction data collection and structure refinement are summarised in **Table S1**. Coordinates and structure factors have been deposited in the Protein Data Bank (PDB) under accession numbers 6H9E and 6H9F.

Active site cavities around the Ado-ligands and the tartrate ion were analysed using the program CavMan employing a modified LIGSITE algorithm[12] with a cutoff-value of 5. The program is available as a PyMOL-plugin from Innophore (www.innophore.com).

All structural figures were prepared using the program PyMOL (www.pymol.org).

Supplementary Tables

Table S1: Summary of crystallographic data.

	GM-AdoMeCbl	GM-AdoEtCbl
beamline	EMBL-BW7B	
temperature	100 K	
wavelength (Å)	0.8428	0.9080
spacegroup	*P*2$_1$	
cell parameters	63.89 Å, 112.62 Å, 108.35 Å<br>β=95.7°	63.75 Å, 112.29 Å, 108.01 Å<br>β=95.7°
resolution range	40.4 - 1.82	35.4 - 2.10
(outer shell)[a]	(1.89 - 1.82)	(2.18 - 2.10)
total reflections	388758 (35193)	337604 (33437)
unique reflections	130639 (12636)	88205 (8790)
multiplicity	3.0 (2.8)	3.8 (3.8)
completeness (%)	96.1 (93.7)	99.9 (99.8)
mean I/σ(I)	19.5 (4.5)	6.7 (2.3)
Wilson B-factor (Å^2)	13.4	10.7
R_{merge}	0.0525 (0.2994)	0.2070 (0.6425)
R_{meas}	0.0635 (0.3658)	0.2410 (0.7488)
R_{pim}	0.0352 (0.2071)	0.1228 (0.3826)
$CC_{1/2}$	0.998 (0.915)	0.974 (0.679)
CC*	1.000 (0.978)	0.993 (0.899)
refl. used in refinement	130639 (12636)	88203 (8777)
refl. used for R_{free}	6647 (599)	4528 (438)
R_{work}	0.1368 (0.2062)	0.1575 (0.2301)
R_{free}	0.1675 (0.2503)	0.2029 (0.2919)
CC_{work}	0.974 (0.936)	0.969 (0.886)
CC_{free}	0.962 (0.903)	0.946 (0.767)
**non-H atoms**	11482	11200
protein	9740	9604
cofactor/ligands	270	242
water	1472	1354
protein residues	1240	1240
**r.m.s.-devs from ideality**		
bond lengths (Å)	0.007	0.007
bond angles (°)	1.17	1.18
**Ramachandran plot**		
favoured (%)	98.0	97.8
allowed (%)	1.5	1.7
outliers (%)	0.5	0.5
rotamer outliers (%)	0.7	0.9
clash score	1.64	3.46
**average B-factor**	14.9	13.5
protein	13.4	12.5
cofactor/ligands	11.5	7.8
water	25.8	21.9
PDB accession code	6H9E	6H9F

[a]Statistics for the highest-resolution shell are shown in parentheses.

Table S2: Geometric parameters of B_{12}-derivatives bound to glutamate mutase. Values are averages of measurements obtained from the two crystallographically independent molecules in the asymmetric unit.

	**AdoCbl**[2c]	**AdoMeCbl**	**AdoEtCbl**
Co-C_ω [Å]	3.17[2]<br>4.19[3]	2.07	1.98
Co…C5′ [Å]	3.17[2]<br>4.19[3]	3.12	4.27
Co-N_{His16} [Å]	2.22	2.21	2.17
Co-C_ω-$C_{\omega-1}$ [°][1]	162[2]	119	113
C_ω-$C_{\omega-1}$-$C_{\omega-2}$ [°]	-	111	108
Co-C_ω-$C_{\omega-1}$-$C_{\omega-2}$ [°]	-	171	-165
C_ω-$C_{\omega-1}$-$C_{\omega-2}$-O/$C_{\omega-3}$ [°]	-	172	-105
ribose conformation	^{2}E, C2′-endo[2]<br>^{3}E, C3′-endo[3]	$^2T_3$, 2,3-twist	E_1, C1′-exo
corrin fold angle [°]	2.1	1.9	3.9
Co-deviation from N_4-plane [Å]	-0.125	-0.008	-0.009

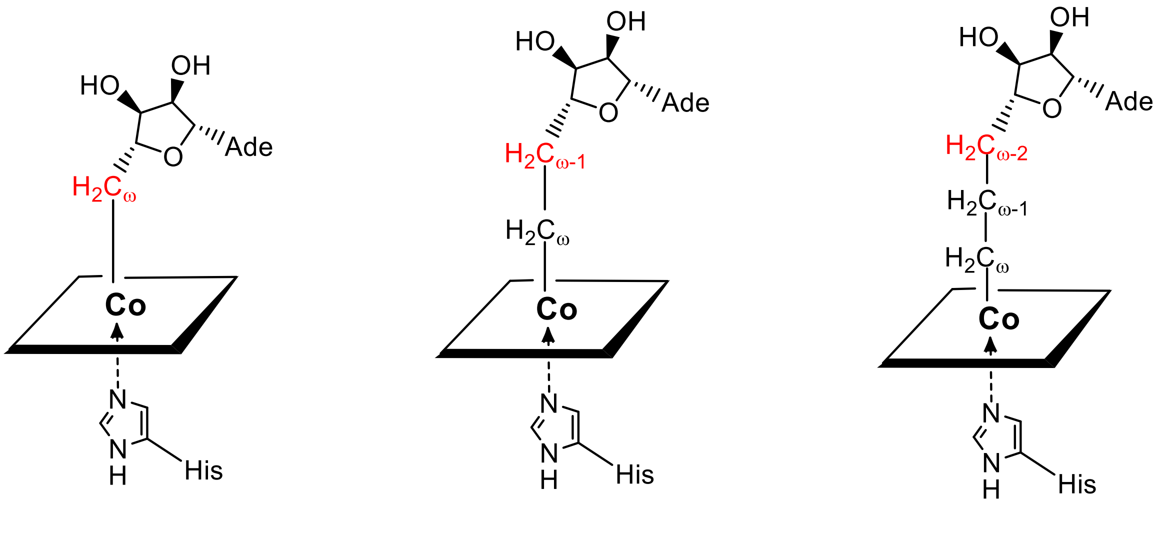

AdoCbl **AdoMeCbl** **AdoEtCbl**

Footnotes:

[1]C_ω: C5′ in GM-AdoCbl, C6′ in GM-AdoMeCbl, C7′ in GM-AdoEtCbl
$C_{\omega-1}$: C4′ in GM-AdoCbl, C5′ in GM-AdoMeCbl, C6′ in GM-AdoEtCbl
$C_{\omega-2}$: C4′ in GM-AdoMeCbl, C5′ in GM-AdoEtCbl
$C_{\omega-3}$: C4′ in GM-AdoEtCbl
O: O4′ in GM-AdoMeCbl
[2]GM-AdoCbl, Ado conformation 1, "radical associated"
[3]GM-AdoCbl, Ado conformation 2, "radical dissociated"

Supplementary Figures

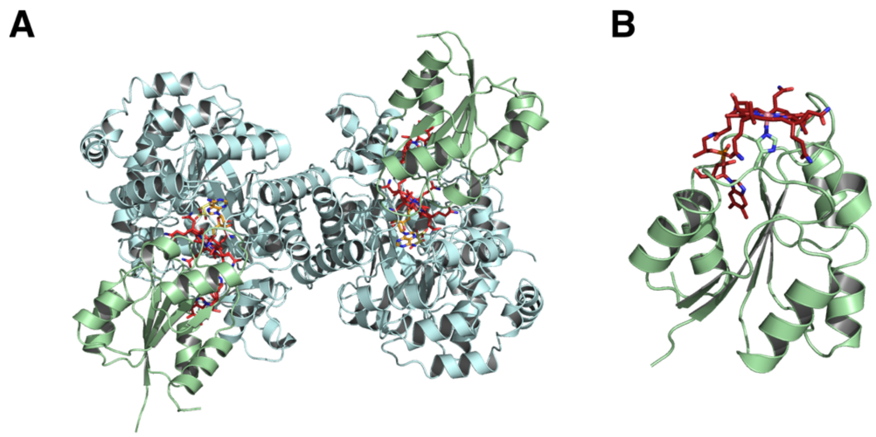

Figure S1: (**A**) Overall structure of GM with the AdoCbl-homologue AdoMeCbl and tartrate bound. The two larger ε-subunits are shown in a cyan cartoon representation, and the smaller σ-subunits are shown in light green. (**B**) Binding of the cobalamin moiety in a "base-off/His-on mode" to the σ-subunit of GM with residue H16 coordinating to the cobalt centre.

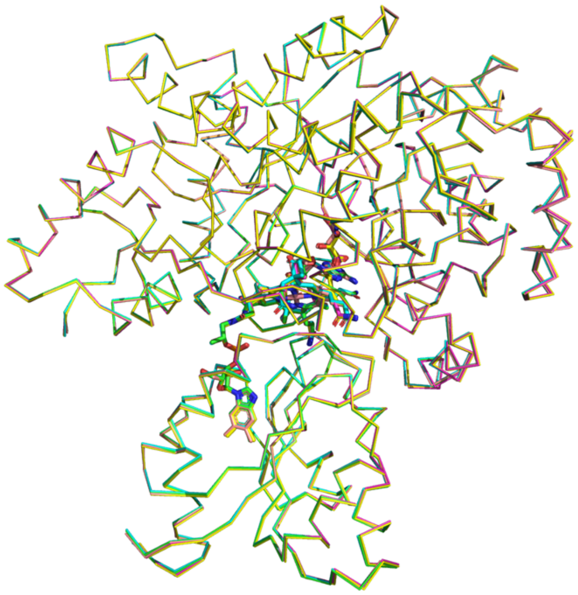

Figure S2: Superposition of the structures of GM reconstituted with CNCbl[2b] (PDB-code: 1CCW, magenta), MeCbl[2b] (PDB-code: 1CB7, yellow), AdoCbl[2c] (PDB-code: 1I9C, pink), AdoMeCbl (PDB-code: 6H9E, green), and AdoEtCbl (PDB-code: 6H9F, cyan). Pairwise Cα-root-mean-square deviations after superimposing the complete tetrameric structures were between 0.1 and 0.2 Å. Only the structures of the respective εσ-heterodimers are shown as ribbon-representations, the bound B_{12}-cofactor molecules and the (pseudo)substrates are depicted as sticks.

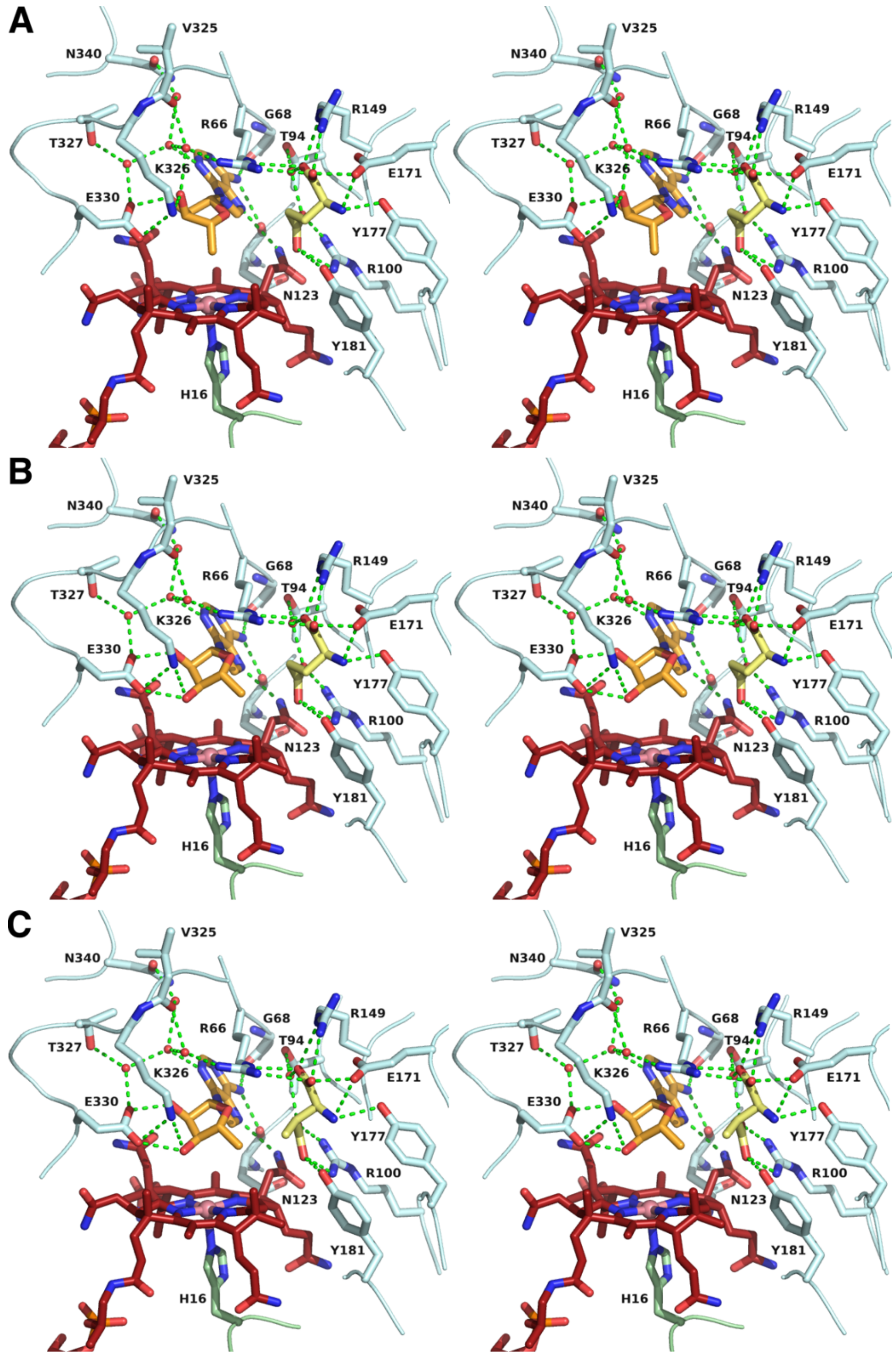

Figure S3: Stereo-views of the active site of GM-AdoCbl (PDB-code: 1I9C)[2c] showing different combinations of Ado-conformation and bound substrate: (**A**) r_a-conf/(*S*)-glutamate, (**B**) r_d-conf/(*S*)-glutamate, and (**C**) r_d-conf/(2*S*,3*S*)-3-methylaspartate. The combination r_a-conf/(2*S*,3*S*)-3-methylaspartate is shown in **Figure 4** of the main manuscript. Display styles and colouring schemes are the same as in the latter figure.

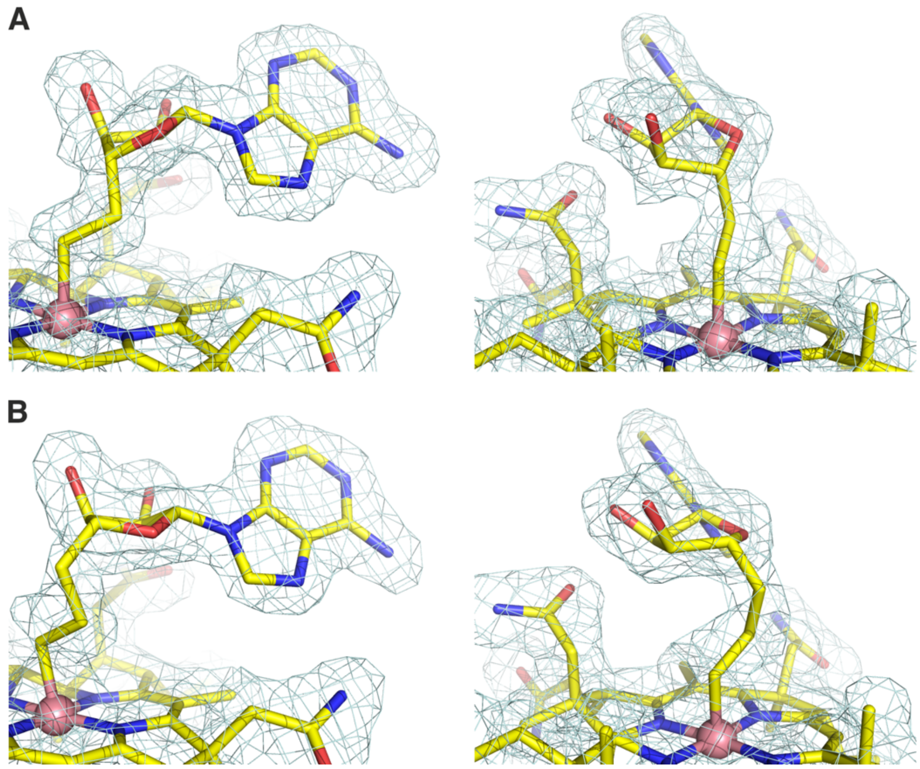

Figure S4: Polder omit-maps[13] contoured at 3σ, calculates around AdoMe (**A**) and AdoEt (**B**) in the respective GM structures. Both structures are shown in two orientations roughly related to each other by a 90°-rotation around the vertical axis. The ligands are shown as sticks, and the difference electron density is depicted as the usual light blue “chicken wire”.

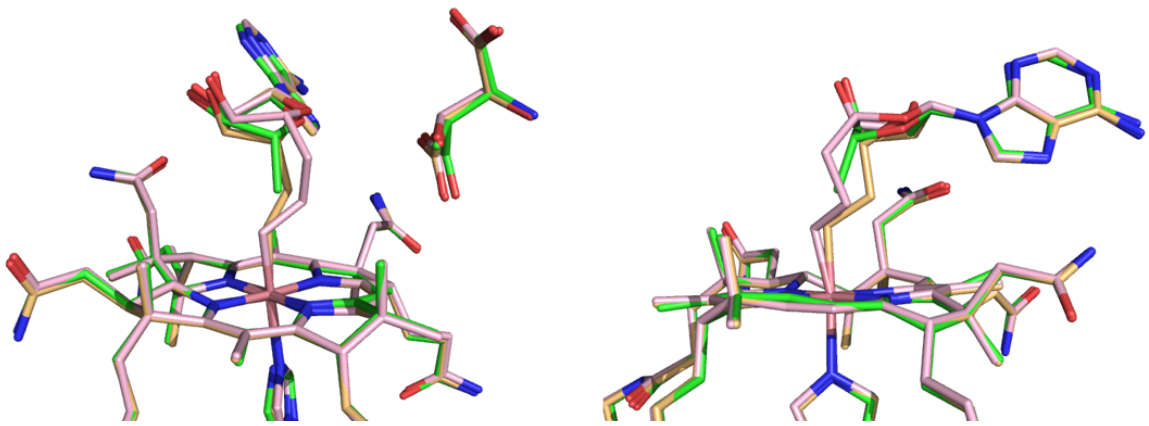

Figure S5: Structures of AdoCbl[2c] (PDB-code: 1I9C, r_a-conf, green), AdoMeCbl (PDB-code: 6H9E, orange) and AdoEtCbl (PDB-code: 6H9F, pink) bound to GM. The four corrin N-atoms were used for the superposition. The molecules are shown in a sticks-representation. The two views are related by an approximately 90°-rotation around the vertical axis.

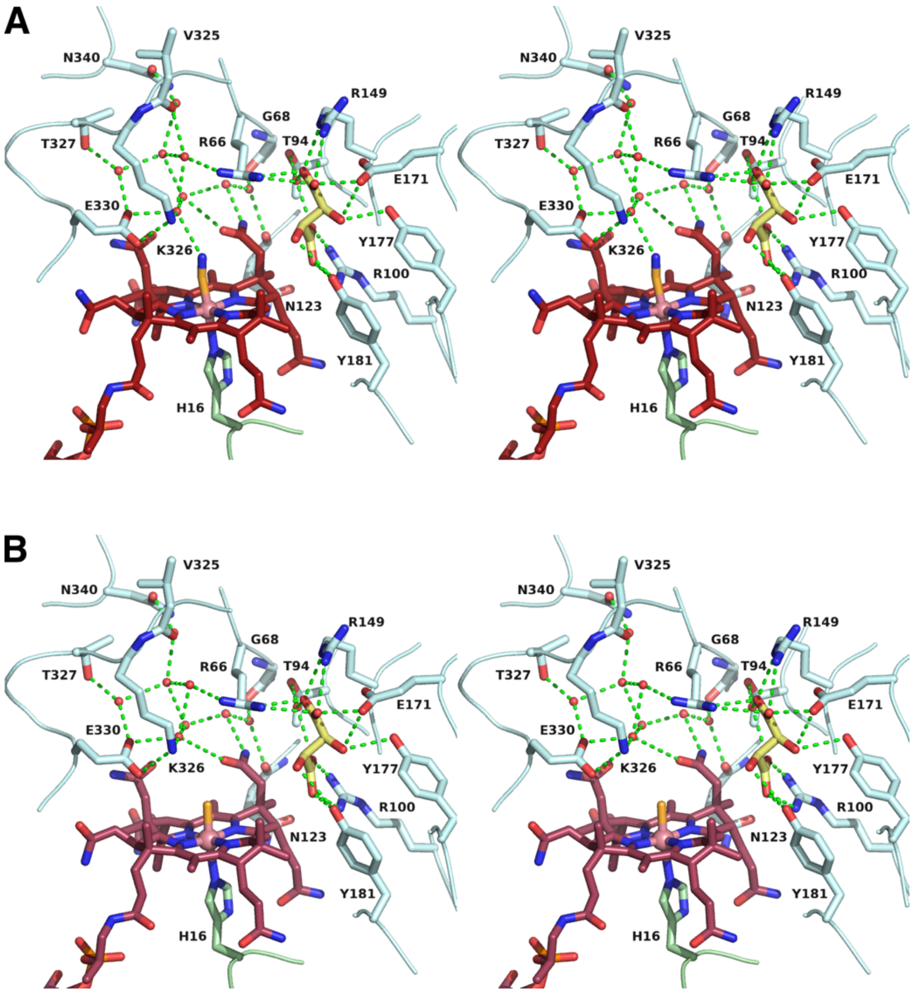

Figure S6: Stereo representation of the active sites of GM reconstituted with CNCbl[2b] (**A**, PDB-code: 1CCW) or MeCbl[2b] (**B**, PDB-code: 1CB7). Display styles and colouring schemes: Amino acid side chains, cofactors, and ligands are shown as sticks. Portions of the main chain of GM are shown as a ribbons-representation. Residues from the ε- and the σ-subunits are shown with light cyan or light green carbon atoms, respectively. The cobalamin is shown in dark red, with the “upper” ligand shown in orange. Tartrate and methylaspartate molecules are coloured yellow. Water molecules are depicted as small red spheres. Hydrogen bonding interactions are shown as green dashed lines (as also used in **Figure S3** and **Figure 4** in the main manuscript).

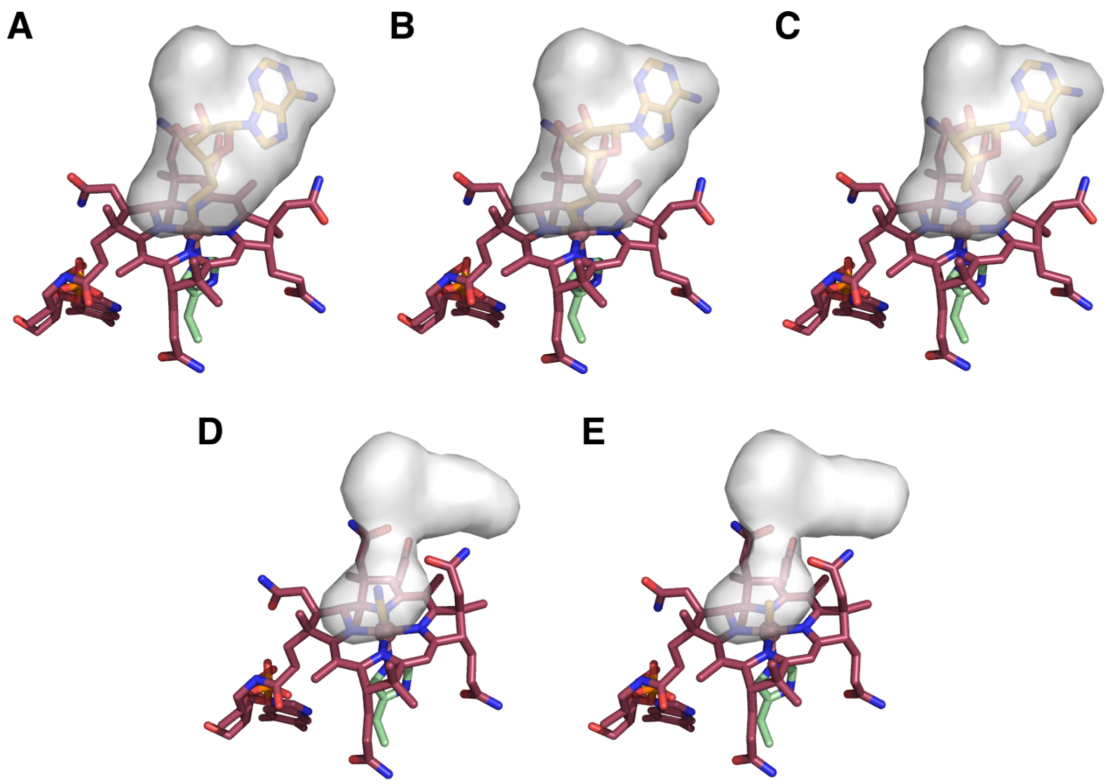

Figure S7: Adenosine binding cavity (ABC) above the northern hemisphere of B_{12} in the structures of GM-AdoMeCbl (**A**), GM-AdoEtCbl (**B**), GM-AdoCbl[2c] (**C**), GM-CNCbl[2b] (**D**), and GM-MeCbl[2b] (**E**). The base-off Cbl is shown in dark red, and the axial ligands are shown in orange ("upper" ligands) and light green (H16 from the σ-subunit of GM), respectively. The cavity was calculated using CavMan (innophore.com) and is depicted as a white semi-transparent surface.

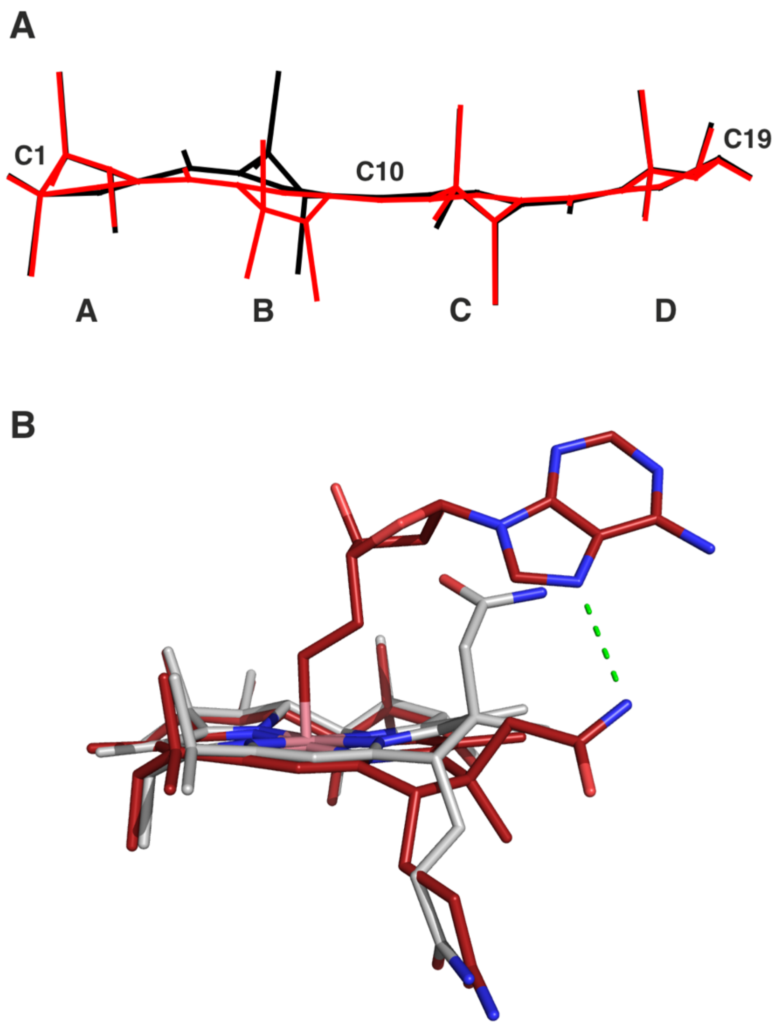

Figure S8: (**A**) Cylinder projection of the corrin ring of B_{12} as observed in the structures of GM reconstituted with CNCbl[2b] (black) and AdoMeCbl (red). The positions of C1, C10, and C19 and the names of the pyrrole rings are given as references. (**B**) Structural superposition (using the four corrin nitrogen atoms) of the cofactor molecules in the structures of GM reconstituted with CNCbl[2b] (grey) and AdoMeCbl (dark red). The H-bond between N3 of the adenine moiety and the amide-NH_2 of the corrin c-sidechain is indicated as a green dashed line.

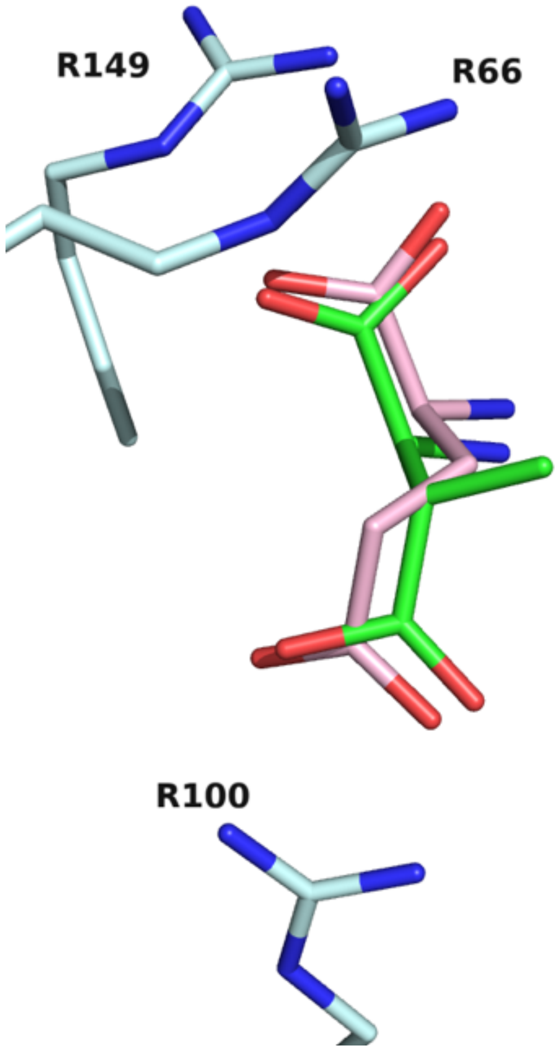

Figure S9: Superposition of (*S*)-glutamate (pink) and (2S,3S)-3-methylaspartate (green) in the structure of GM-AdoCbl (PDB-code: 1I9C)[2c] viewed from the side. The three arginine residues forming the “arginine claw” are labelled.

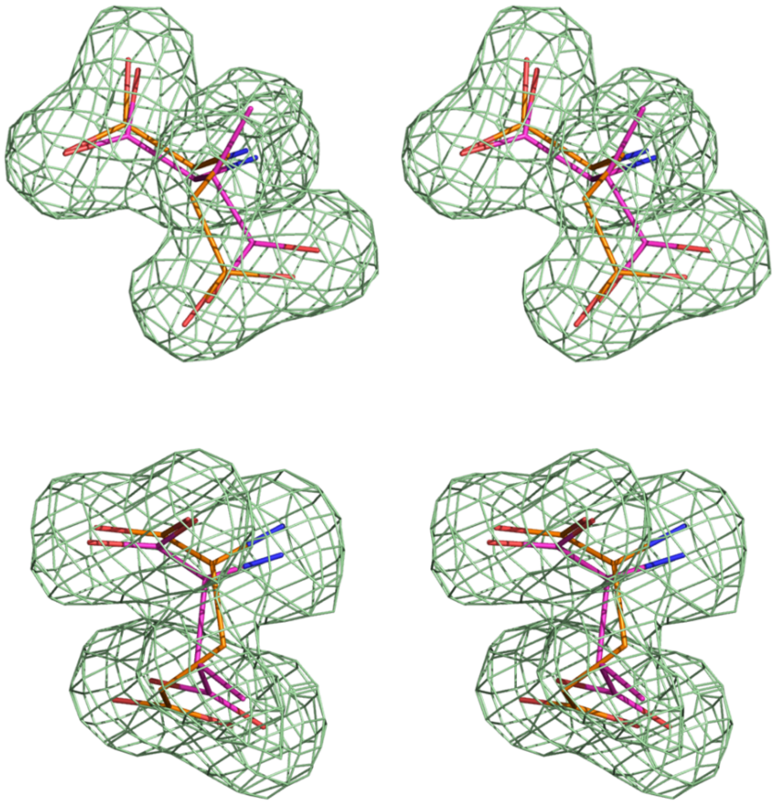

Figure S10a: Stereo-views of the electron density (Polder omit-maps[13] contoured at 3σ, green mesh) for (*S*)-glutamate (orange) and (2*S*,3*S*)-3-methylaspartate (magenta) in the structure of GM-AdoCbl (PDB-code: 1I9C),[2c] viewed from the side (top) and from above (bottom). For the map calculations, coordinates and diffraction data were retrieved from the PDB-REDO server.[14]

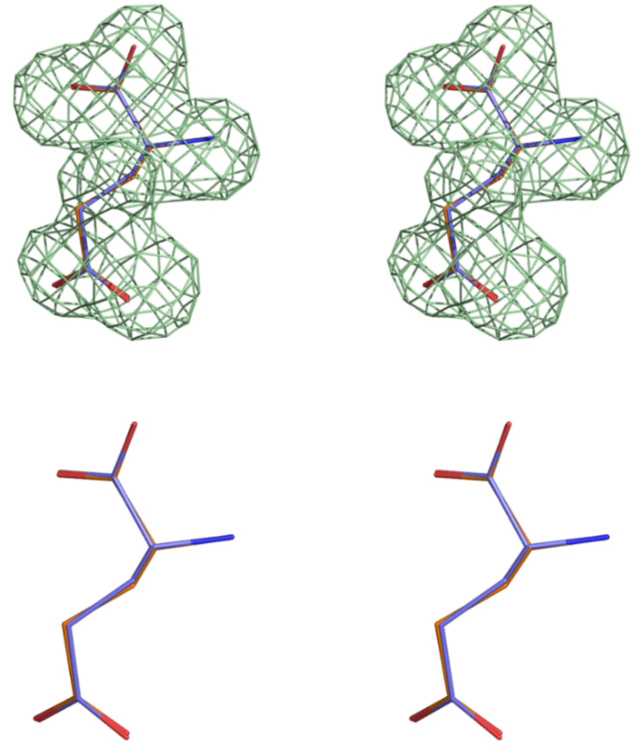

Figure S10b: Structural differences between glutamate (orange) and 4-glutamylradical (blue) when refined against the GM-AdoCbl data.[2c] The same orientation is shown with (top) and without (bottom) Polder omit-map.[13] For the refinement, coordinates and diffraction data were retrieved from the PDB-REDO server.[14]

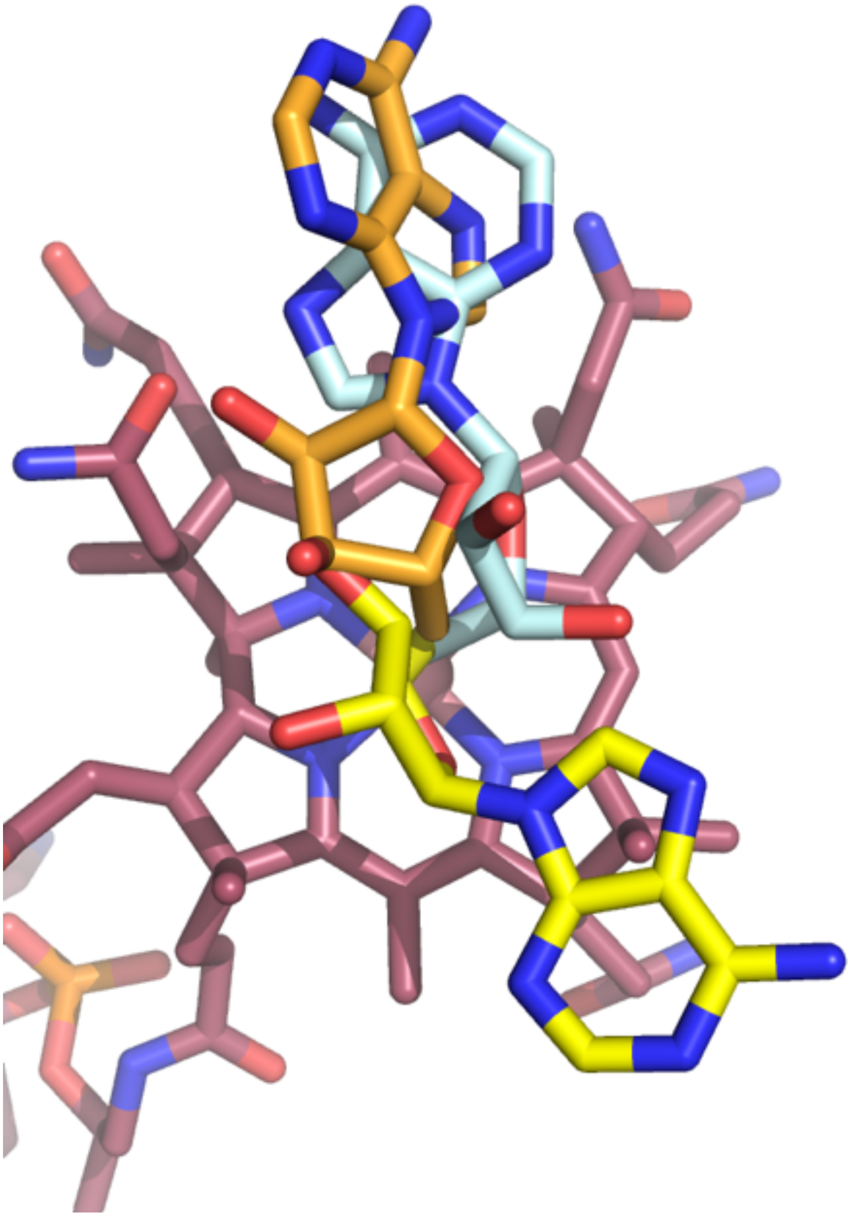

Figure S11: Positions of the adenosyl ligand relative to the corrin ring in GM-AdoCbl (r_a-conf, orange, code: 1I9C),[2c] MCM from *Mycobacterium tuberculosis* (cyan, PDB-entry: 6OXC),[15] and in crystalline coenzyme B_{12} (yellow; CSD-entry: FIZMUW).[16]

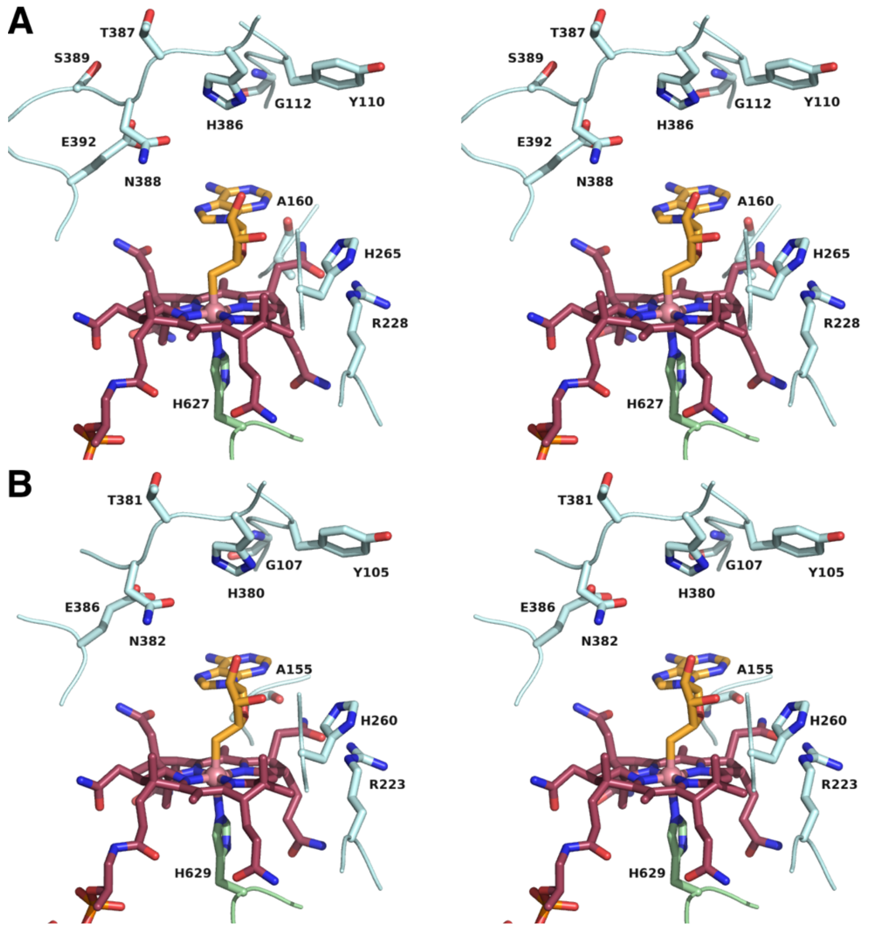

Figure S12: Stereo-views of the regions around the Ado-ligand in the structures of substrate-free MCM from humans (PDB-code: 2XIJ)[17] (**A**) and *Mycobacterium tuberculosis* (PDB-code: 6OXC)[15] (**B**). The structures were re-refined starting from structures obtained from the PDB-REDO server[14] and employing equivalent restraints for the Co-C bond as in the refinements of GM-AdoMeCbl and GM-AdoEtCbl. Display styles and colouring schemes are the same as in **Figures S3** and **S6**, as well as in **Figure 4** in the main manuscript.

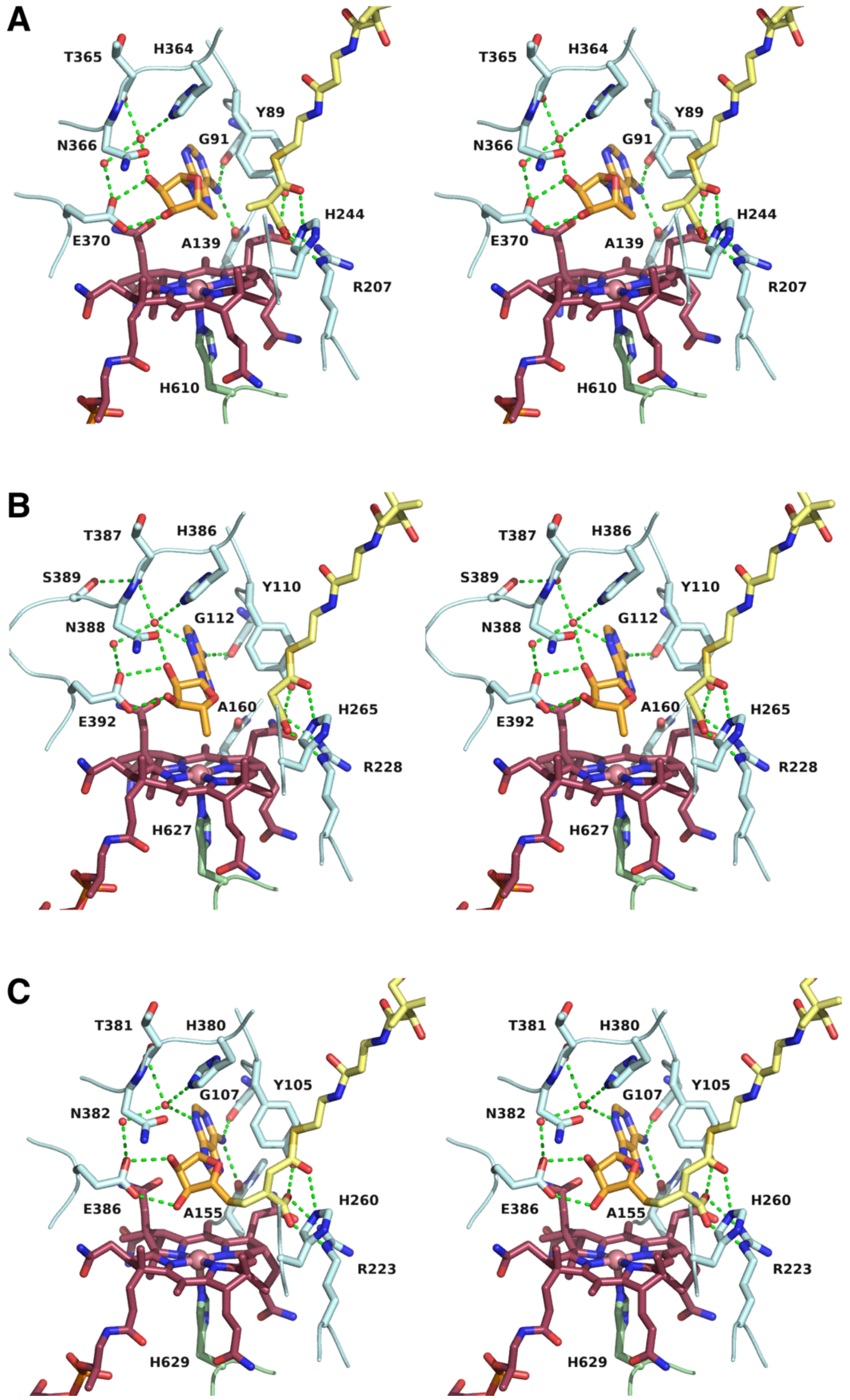

Figure S13a: Stereo-views of active sites of acyl-CoA mutases (for details, see next page).

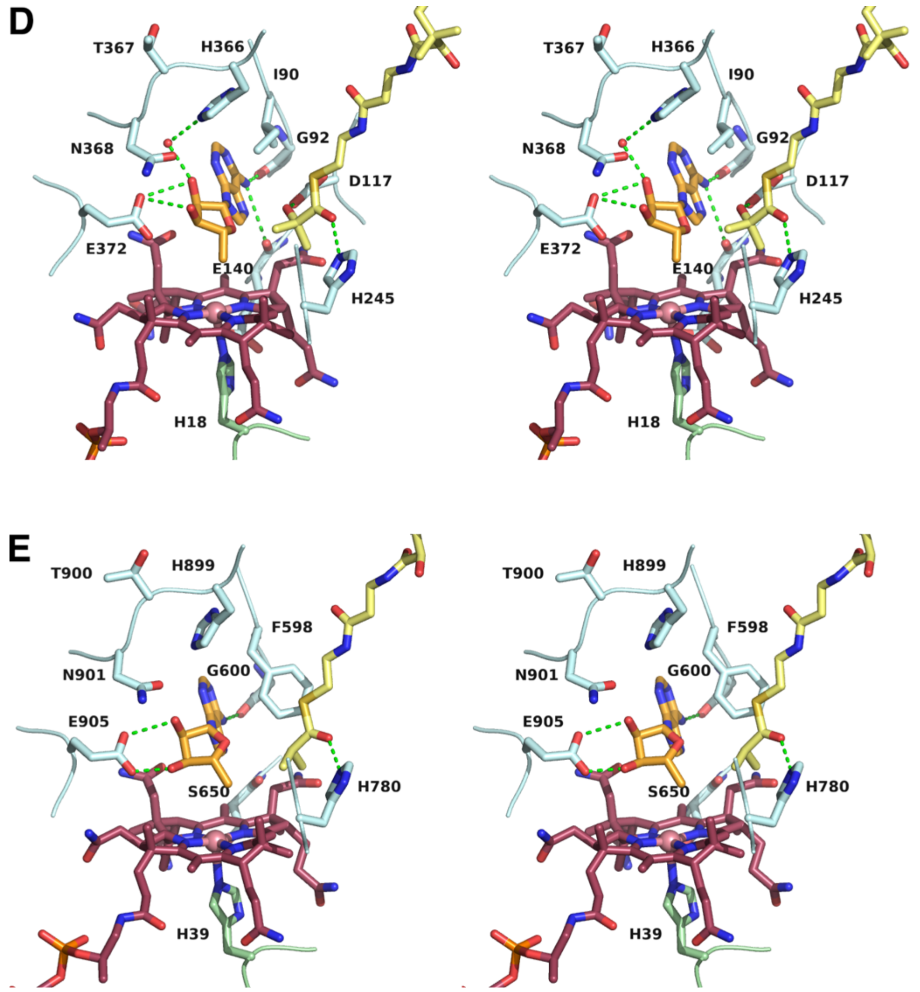

Figure S13b: Stereo-views of active sites of acyl-CoA mutases. (**A**) MCM from *Propionibacterium shermanii* (PDB-code: 4REQ),[18] (**B**) human MCM (PDB-code: 2XIQ),[17] (**C**) MCM from Mycobacterium tuberculosis (PDB-code: 6OXD),[15] (**D**) 2-hydroxyisobutyryl-CoA mutase from *Aquincola tertiaricarbonis* (PDB-code: 4R3U),[19] and (**E**) iso-butyryl-CoA-mutase from *Cupriavidus metallidurans* CH3 (PDB-code: 5CJT).[20] The structures were obtained from the PDB-REDO server.[14] Display styles and colouring schemes are the same as in **Figures S3**, **S6** and **S12**, as well as in **Figure 4** in the main manuscript.

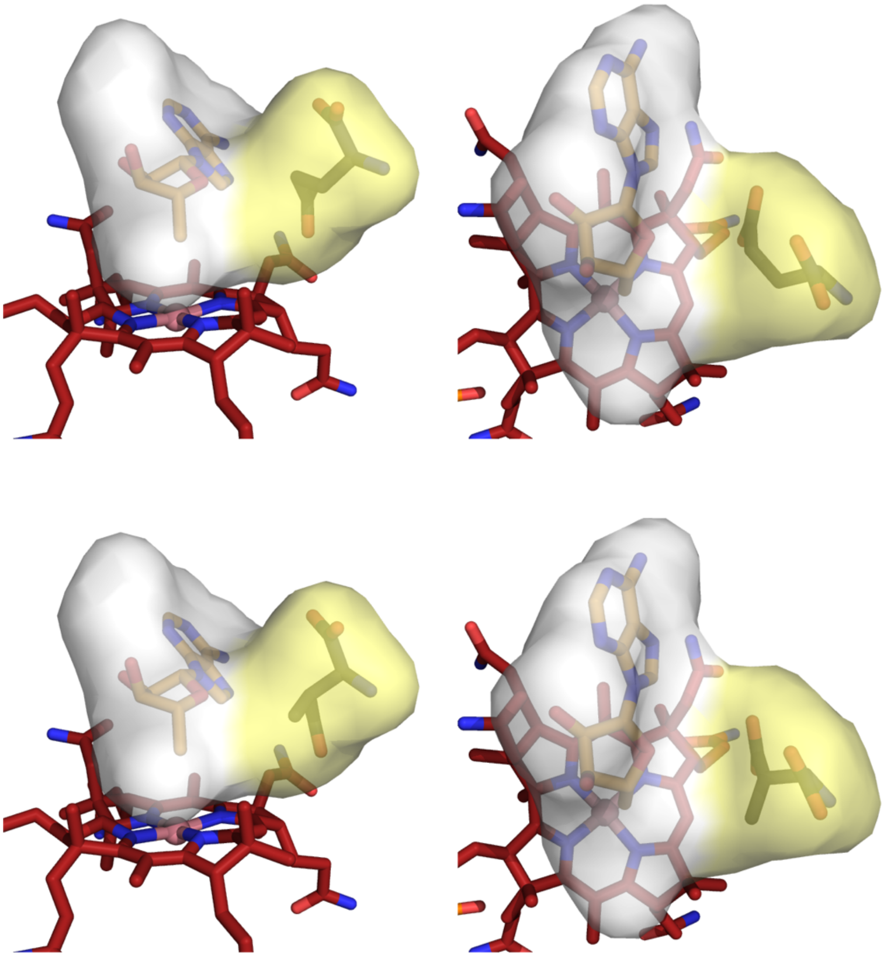

Figure S14: Caged radical reaction space (CSSR) next to the bound B_{12}-cofactor in GM-AdoCbl (PDB-code: 1I9C)[2c] showing the Ado-moiety in the r_a-conformation, and with the substrates (*S*)-glutamate (top) or with (2*S*,3*S*)-3-methylaspartate (bottom) bound. The cavities were calculated using CavMan (innophore.com). The colouring scheme is the same as in **Figure 7** in the main manuscript.

Supplementary References

[1] a) S. Gschösser, R. B. Hannak, R. Konrat, K. Gruber, C. Mikl, C. Kratky, B. Kräutler, *Chem. Eur. J.* **2005**, *11*, 81-93; b) R. B. Hannak, PhD thesis, University of Innsbruck **1996**.
[2] a) R. Reitzer, M. Krasser, G. Jogl, W. Buckel, H. Bothe, C. Kratky, *Acta Crystallogr. D* **1998**, *54*, 1039-1042; b) R. Reitzer, K. Gruber, G. Jogl, U. G. Wagner, H. Bothe, W. Buckel, C. Kratky, *Structure* **1999**, *7*, 891-902; c) K. Gruber, R. Reitzer, C. Kratky, *Angew. Chem. Int. Ed.* **2001**, *40*, 3377-3380.
[3] G. Winter, *J. Appl. Crystallogr.* **2010**, *43*, 186-190.
[4] T. G. G. Battye, L. Kontogiannis, O. Johnson, H. R. Powell, A. G. W. Leslie, *Acta Crystallogr. D* **2011**, *67*, 271-281.
[5] W. Kabsch, *Acta Crystallogr. D* **2010**, *66*, 125-132.
[6] P. Evans, *Acta Crystallogr. D* **2006**, *62*, 72-82.
[7] M. D. Winn, C. C. Ballard, K. D. Cowtan, E. J. Dodson, P. Emsley, P. R. Evans, R. M. Keegan, E. B. Krissinel, A. G. W. Leslie, A. McCoy, S. J. McNicholas, G. N. Murshudov, N. S. Pannu, E. A. Potterton, H. R. Powell, R. J. Read, A. Vagin, K. S. Wilson, *Acta Crystallogr. D* **2011**, *67*, 235-242.
[8] P. D. Adams, P. V. Afonine, G. Bunkóczi, V. B. Chen, I. W. Davis, N. Echols, J. J. Headd, L. W. Hung, G. J. Kapral, R. W. Grosse-Kunstleve, A. J. McCoy, N. W. Moriarty, R. Oeffner, R. J. Read, D. C. Richardson, J. S. Richardson, T. C. Terwilliger, P. H. Zwart, *Acta Crystallogr. D* **2010**, *66*, 213-221.
[9] P. Emsley, B. Lohkamp, W. G. Scott, K. Cowtan, *Acta Crystallogr. D* **2010**, *66*, 486-501.
[10] G. J. Kleywegt, A. T. Brunger, *Structure* **1996**, *4*, 897-904.
[11] C. J. Williams, J. J. Headd, N. W. Moriarty, M. G. Prisant, L. L. Videau, L. N. Deis, V. Verma, D. A. Keedy, B. J. Hintze, V. B. Chen, S. Jain, S. M. Lewis, W. B. Arendall, J. Snoeyink, P. D. Adams, S. C. Lovell, J. S. Richardson, D. C. Richardson, *Protein Sci.* **2018**, *27*, 293-315.
[12] M. Hendlich, F. Rippmann, G. Barnickel, *J. Mol. Graphics Model.* **1997**, *15*, 359-363.
[13] D. Liebschner, P. V. Afonine, N. W. Moriarty, B. K. Poon, O. V. Sobolev, T. C. Terwilliger, P. D. Adams, *Acta Crystallogr. D* **2017**, *73*, 148-157.
[14] R. P. Joosten, F. Long, G. N. Murshudov, A. Perrakis, *IUCrJ* **2014**, *1*, 213-220.
[15] M. Ruetz, G. C. Campanello, M. Purchal, H. Shen, L. McDevitt, H. Gouda, S. Wakabayashi, J. Zhu, E. J. Rubin, K. Warncke, V. K. Mootha, M. Koutmos, R. Banerjee, *Science* **2019**, *366*, 589-593.
[16] H. F. J. Savage, P. F. Lindley, J. L. Finney, P. A. Timmins, *Acta Crystallogr. B* **1987**, *43*, 280-295.
[17] D. S. Froese, G. Kochan, J. R. C. Muniz, X. Wu, C. Gileadi, E. Ugochukwu, E. Krysztofinska, R. A. Gravel, U. Oppermann, W. W. Yue, *J. Biol. Chem.* **2010**, *285*, 38204-38213.
[18] F. Mancia, P. R. Evans, *Structure* **1998**, *6*, 711-720.
[19] N. Kurteva-Yaneva, M. Zahn, M. T. Weichler, R. Starke, H. Harms, R. H. Müller, N. Sträter, T. Rohwerder, *J. Biol. Chem.* **2015**, *290*, 9727-9737.
[20] M. Jost, D. A. Born, V. Cracan, R. Banerjee, C. L. Drennan, *J. Biol. Chem.* **2015**, *290*, 26882-26898.
